# Circulating anti-inflammatory adipokines High Molecular Weight Adiponectin and Zinc-α2-glycoprotein (ZAG) are inhibited in early sepsis, but increase with clinical recovery: a pilot study

**DOI:** 10.1186/1471-2253-14-124

**Published:** 2014-12-18

**Authors:** Ingeborg D Welters, Chen Bing, Cherlyn Ding, Martin Leuwer, Alison M Hall

**Affiliations:** Department of Obesity and Endocrinology, Institute of Ageing and Chronic Disease, University of Liverpool, Duncan Building, Daulby Street, L69 3GA Liverpool, UK; Intensive Care Unit, Royal Liverpool University Hospital, Liverpool, UK; Department of Molecular and Clinical Pharmacology, Institute of Translational Medicine, University of Liverpool, Liverpool, UK

**Keywords:** Adiponectin, HMW adiponectin, Zinc-α2-glycoprotein, Sepsis, Adipokines

## Abstract

**Background:**

Adipose tissue has been identified as an endocrine organ secreting adipokines involved in metabolic and inflammatory pathways. Adiponectin, an anti-inflammatory adipokine, is reduced in sepsis. High Molecular Weight (HMW) adiponectin, the biologically most relevant molecule, has been investigated very little in human sepsis. Zinc-alpha2-glycoprotein (ZAG) is a novel adipokine and its expression in adipose tissue is positively correlated with adiponectin expression. It is not yet known whether ZAG has a role in sepsis. In this study we assessed levels of HMW adiponectin and ZAG during different stages of sepsis.

**Methods:**

A prospective observational pilot study was carried out on 21 septic patients. Serum samples were taken on day 1 and 2 post ICU admission and on day of discharge. Samples were analysed for total and HMW adiponectin, HMW/total adiponectin ratio, and ZAG. Results were correlated with clinical and metabolic data.

**Results:**

There were no differences in total adiponectin, HMW adiponectin and ZAG plasma concentrations between day 1 (admission) and day 2 of the sepsis episode. Compared to admission, a significant increase in total and HMW adiponectin and ZAG was observed on the day of discharge when clinical improvement had been achieved. There was also an increase in the HMW/total adiponectin ratio at that time.

**Conclusions:**

Our data demonstrate an increase in both HMW adiponectin and total adiponectin in patients who had clinically recovered from sepsis. The increase in HMW/total adiponectin ratio with improvement of the clinical condition suggests that HMW adiponectin may have a greater role in the inflammatory process and insulin resistance seen in sepsis. In this pilot study, we have also demonstrated a significant increase in ZAG in critically ill patients temporally related to recovery from sepsis.

## Introduction

In the last 15 years, white adipose tissue (WAT) has been identified as a sophisticated endocrine organ secreting adipokines with a myriad of different functions including involvement in metabolic, inflammatory and proliferative pathways [1]. Whilst most adipokines possess pro-inflammatory properties, adiponectin and Zinc-α2-glycoprotein (ZAG) have been proposed as potential anti-inflammatory mediators released from WAT [1, 2].

ZAG, a soluble protein first isolated from human plasma, has originally been regarded a cancer marker, since its levels are elevated in patients with prostate and cervical cancer [2]. However, ZAG is also expressed in WAT and secreted by adipocytes [3, 4]. Expression and secretion of ZAG by human adipocytes is suppressed by macrophage-derived factors and TNF-α, paralleling release patterns for adiponectin [5]. Gene expression of adiponectin and ZAG in WAT is down-regulated in obesity. ZAG also increases adiponectin production in human adipocytes [6] and increases lipid loss in cachexia by stimulating lipolysis [7]. Cachexia is common during and after critical illness, suggesting a potential role for ZAG. However, changes in ZAG concentrations in sepsis have not been studied to date.

Adiponectin is now considered the major anti-inflammatory adipokine produced by WAT [8]. Its numerous actions include reduction of the phagocytic activity of macrophages and inhibition of the production of inflammatory cytokines from macrophages and adipose tissue [9–16]. Adipocytes release different oligomeric isoforms of adiponectin, including trimeric, hexameric and the high molecular weight (HMW) oligomeric complex [17]. HMW adiponectin is postulated as the more biologically active molecule, binding avidly to its receptors, thus stimulating AMP activated protein kinase [18–20].

We have previously demonstrated that total adiponectin production is reduced in mice following treatment with lipopolysaccharide, suggesting that adiponectin is actively involved in the inflammatory response of WAT [21]. Recent clinical studies have shown that circulating concentrations of total adiponectin are low in the acute phase of sepsis and increase with recovery [22]. To date no clinical studies have investigated the role of HMW adiponectin in relation to total adiponectin concentrations in acute life-threatening infection.

We therefore aimed to investigate the serum concentrations of high molecular weight adiponectin and its relation to total circulating adiponectin in septic patients. To evaluate whether further anti-inflammatory adipokines undergo similar changes during acute phase sepsis and clinical recovery we also determined serum levels of ZAG.

## Methods

A prospective observational study was carried out to determine the plasma concentrations of total and HMW adiponectin in septic patients. Ethical approval was received from the Local Research and Ethics committee (Liverpool Paediatric Research Ethics Committee 06/Q1502/7) and from the NHS trust (no 3258) allowing witnessed assent from relatives with patients being informed as soon as practical to obtain retrospective consent.

### Patient recruitment

21 patients (18–85 years) admitted to the Intensive Care Unit (ICU) at the Royal Liverpool University Hospital with sepsis or septic shock according to ACCP /SCCM Consensus Conference guidelines were recruited into the study [23].

Exclusion criteria were pregnancy/lactation, insulinoma, immunosuppression due to other causes than sepsis (post organ transplant, AIDS, ongoing chemotherapy), and <18 years of age. Demographic, clinical and laboratory data were collected. Data relating to ongoing therapies (inotrope and insulin requirements, feeding regime, and daily glucose and lactate measurements) were also collected. Serum samples were taken from each patient on admission to ICU. Further serum samples were taken on day 2 and on day of discharge. For patients who died, only day 1 and day 2 samples were taken. Following centrifugation (10 minutes at 3000 rpm), serum was obtained, aliquoted and stored at −80 C until analysis.

### Sample analysis

#### Total adiponectin ELISA

(R&D Systems DY1065): Samples were homogenized in lysis buffer (sucrose 250 mM/HEPES 1 mM/EDTA 0.2 mM, pH 7.2). Capture antibody (2 μg/ml) was used to coat a 96 well plate. Plates were blocked using 1% Bovine Serum Albumin (BSA) in Phosphate-buffered Saline (PBS). Samples and standards (diluted in 1% BSA in PBS) were added to the plate and incubated. After washing, detection antibody (concentration 2 μg/ml) was added to each well and following incubation, Streptavadin-HRP (concentration 1/200) was added. Colour substrate solution (1:1 mixture of Colour Reagent A (H_2_O_2_) and Colour Reagent B (Tetramethylbenzidine, 1:1 dilution) was added and the reaction stopped using stop solution (2NH_2_SO_4_). Optical density was determined using a microplate reader at dual wavelength 450 nm with a reference wavelength 570 nm.A high standard of 4000 pg/ml was used with serial dilutions.

#### High molecular weight adiponectin

(ALPCO, Salem, USA): All measurements were performed according to the manufacturer’s instructions. In brief, samples were pre-treated with Protease II and the remaining HMW fraction was treated with sample pre-treatment buffer. Protease I was combined with the sample and incubated at 37 C. Immediately, sample pre-treatment buffer was added, vortexed and diluted. 50 μl of each standard diluted pre-treated sample was added to the appropriate wells and incubated at room temperature. Following washing, biotin labelled monoclonal antibody was added to each well and incubated. Following a second wash, enzyme labelled streptavidin was added to each well and incubated. Subsequently, substrate solution was added to each well and following incubation, the reaction was terminated using stop reagent. The absorbance of each well was measured using a microplate reader set to 492 nm, with a reference wavelength of 600–700 nm.

#### Zinc-α2-glycoprotein (ZAG)

(BioVendor, Laborartorni Medicina a.s, Czech Republic): All measurements were performed according to the manufacturer’s instructions. In brief, 100 μl of diluted standards, quality controls, dilution buffer and diluted samples, were pipetted into a 96-microtitre plate in duplicate. The plate was incubated at room temperature for 1 hour after three washes, 100 μl of Conjugate Solution was pipetted into each well and incubated at room temperature for a further 1 hour. After three washes, 100 μl of substrate solution was added into each well and incubated for 10 minutes at room temperature. Reactions were stopped using 100 μl of Stop Solution and the absorbance of each well was determined using a microplate reader set to 450 nm with reference wavelength of 630 nm.

### Statistical analysis

Descriptive data are presented as mean and standard deviation or as median and interquartile range as indicated. Categorical parameters are given in cross tables. For graphic representation boxplots were used. All data were examined for normal distribution using normal Q-Q plots and the Shapiro-Wilk test. Since the majority of data were not normally distributed the nonparametric Mann–Whitney U-Test was used for all analyses comparing two groups. The Chi-squared test was used to determine any difference in categorical variables. The non-parametric Wilcoxon test for paired data was used to evaluate changes in time course. Correlations between ZAG and adiponectin changes were assessed by calculating Spearman’s and Pearson’s correlation coefficient as indicated.

Results are displayed as median and interquartile range unless stated otherwise. Adjustments for multiple testing were performed using Bonferroni’s procedure as indicated. All statistics were performed with R version 2.8.0. p < 0.05 was considered significant.

## Results

### Biometric, clinical and laboratory data

Twenty-one patients with severe sepsis were recruited into the study. Eight patients presented with abdominal sepsis, 9 patients with chest sepsis, 1 patient with necrotising fasciitis, 2 patients with osteomyelitis and 1 patient with urinary sepsis. For 10 patients follow-up blood samples were not available (5 patients died, 2 patients were discharged on day 2 and in 3 patients follow-up blood samples for adipokine determination could not be obtained). 10 patients were female and 11 were male.

Baseline clinical, laboratory and metabolic data are shown in Tables 1 and 2. Median age was 63 years (55–71 years) with a median APACHE II score of 20. Clinically, patients were not universally pyrexial but were tachycardic and tachypneoic, all requiring more than 50% supplemental oxygen. None of the patients required blood products. Biochemical and haematological markers (White cell count and C-Reactive Protein (CRP)) were elevated and most had a degree of renal impairment. 9 of the 21 patients had to be started on renal replacement therapy for acute kidney injury. There was a positive correlation of total adiponectin and severity of illness scores (Day 1: r = 0.50, p < 0.01, Day 2: r = 0.42, p < 0.01, Day of discharge 0.35, p < 0.05) as determined by Spearman’s correlation coefficient. There was no significant correlation between HMW adiponectin and APACHE II score. No significant differences in total and HMW adiponectin or ZAG were observed between patients who survived and those who died. There were also no differences in biometric data (age, gender, BMI) and severity of disease (APACHE II).

**Table 1:** **Biometric and clinical data on admission, day 1 and day of discharge**

	n	Median	Range	Mean ± SD	95% CI
Age (years)	21	63	[55; 71]	62.2 ± 14.1	[55.84; 68.64]
APACHE II	21	20	[18; 24]	20.8 ± 6.5	[17.86; 23.76]
Temp (°C)	19	37.1	[36.95; 38.15]	37.2 ± 1.4	[36.57; 37.91]
FiO_2_	21	0.6	[0.5; 0.8]	0.6 ± 0.2	[0.53; 0.75]
Height (cm)	21	167	[162; 176]	167.9 ± 9.6	[163.5; 172.29]
Weight (kg)	21	84.4	[71; 93]	81.5 ± 16.3	[74.08; 88.94]
BMI (kg.m^2^)	21	30	[24; 32.08]	29.1 ± 6.6	[26.1; 32.13]
LOS (days)	21	7	[3; 15]	9.3 ± 8.4	[5.46; 13.12]

Biometric data for all patients including age, severity of illness scoring (APACHE II), height, weight and length of stay (LOS) in the ICU. Parameters are displayed as median, range (IQR), mean ± standard deviation (SD) and 95% confidence intervals (CI) of means (APACHE: acute physiology and chronic health evaluation, BMI: Body mass index).

**Table 2:** **Laboratory results on admission, day 1 and day of discharge**

Admission	n	Median	Range	Mean ± SD	95% CI
**WCC (x10/L)**	21	23	[11.7; 31.9]	21.7 ± 12.3	[16.1; 27.3]
**CRP (mg/L)**	18	198	[150.3; 240.5]	198.6 ± 22.2	[152.8; 244.5]
**Hb (g/dL)**	21	10.3	[8.5; 11.2]	10.2 ± 2.2	[9.2; 11.18]
**Creatinine (μmol/L)**	21	114	[76; 183]	153.0 ± 123.2	[96.9; 209.0]
**Urea (mmol/L)**	21	9.2	[7.4; 13.1]	11.4 ± 6.4	[8.5; 14.3]
**Bilirubin (μmol/L)**	20	15.0	[8.0; 34.8]	28.7 ± 30.2	[14.6; 42.9]
**Glucose (mmol/L)**	21	6.65	[5.86; 8.3]	7.3 ± 2.0	[6.4; 8.2]
**Day 2**					
**WCC (x10/L)**	21	22.2	[5.1; 36.6]	22.2 ± 10.3	[17.5; 26.9]
**CRP (mg/L)**	9	234.0	[107.0; 338.0]	236.1 ± 63.5	[284.9; 237.6]
**Hb (g/dL)**	21	9.5	[7.8; 13.5]	9.9 ± 1.7	[9.1; 10.6]
**Creatinine (μmol/L)**	21	126.0	[35.0; 425]	137.0 ± 89.5	[177.8; 127.1]
**Urea (mmol/L)**	21	10.1	[3.4; 33]	11.6 ± 6.2	[8.8; 14.4]
**Bilirubin (μmol/L)**	21	15.0	[3.0; 103.0]	28.8 ± 33.2	[13.6; 43.9]
**Glucose (mmol/L)**	21	7.3	[3.7; 10.7]	7.3 ± 1.5	[6.6; 8.0]
**Day of Discharge**					
**WCC (x10/L)**	18	14.9	[8.6; 30.5]	16.8 ± 7.3	[13.3; 20.3]
**CRP (mg/L)**	6	66.5	[36.0; 199.0]	97.8 ± 67.1	[27.4; 168.3]
**Hb (g/dL)**	18	9.3	[8.0; 10.6]	9.1 ± 0.7	[8.7; 9.6]
**Creatinine (μmol/L)**	18	54.5	[24.0; 285]	89.7 ± 71.5	[54.1; 125.2]
**Urea (mmol/L)**	18	7.6	[2.7; 24.1]	9.2 ± 5.5	[6.5; 12.0]
**Bilirubin (μmol/L)**	18	9.5	[4.0; 430.0]	54.6 ± 110.8	[−0.5; 109.6]
**Glucose (mmol/L)**	18	6.3	[4.7; 9.7]	6.5 ± 1.3	[5.8; 7.1]

Laboratory data obtained on admission to Intensive Care, on day 2 and on day of discharge from Intensive Care. WCC: white cell count, CRP: C-Reactive protein, Hb: Haemoglobin. Parameters are displayed as median, range (IQR), mean ± standard deviation (SD) and 95% confidence intervals (CI) of means. CRP is not routinely measured during Intensive Care stay.

On the day of admission 6 of 21 patients required intravenous insulin infusion to maintain blood glucose levels between 3.5 and 8 mmol/L. There was no significant correlation with average amount of insulin required per hour and weight, total and HMW adiponectin levels, or ZAG levels at any time point.

### Total Adiponectin and HMW adiponectin concentrations

**Figure 1:**
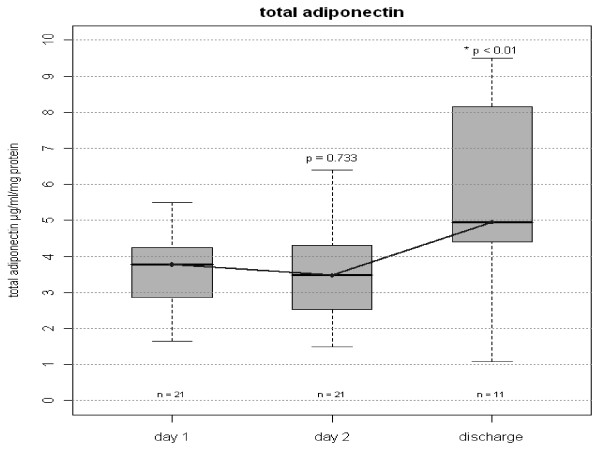
**Total Plasma Adiponectin in on day 1, 2 and discharge.** Serum concentrations of total adiponectin in septic patients. Figure shows median and interquartile range (grey box). Comparisons were made between Day 1 and Day 2 and between Day 1 and Day of Discharge. P values before Bonferroni correction are given in the graph. Corrected p value for Day 2 versus Day of Discharge is <0.02.

**Figure 2:**
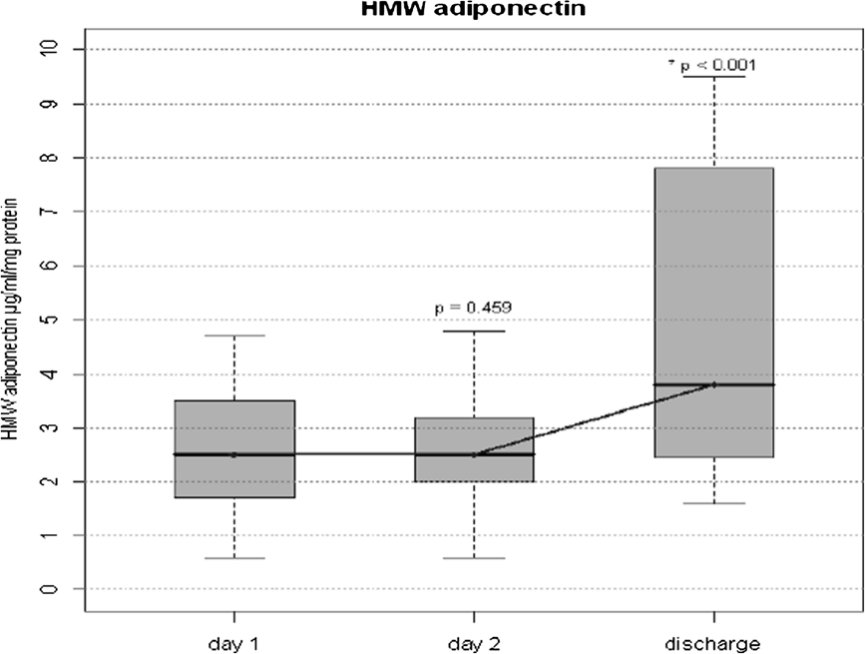
**High molecular weight Plasma Adiponectin on day 1, 2 and discharge.** Serum concentrations of High molecular weight (HMW) adiponectin. Figure shows median and interquartile range (grey box). Comparisons were made between Day 1 and Day 2 and between Day 1 and Day of Discharge. P values before Bonferroni correction are given in the graph. Corrected p value for Day 2 versus Day of Discharge is <0.002.

Total adiponectin as well as HMW adiponectin concentrations were lower during the first two days of the septic episode. A significant increase in total and HMW adiponectin was observed from day 1 (admission) to day of discharge when the clinical condition had improved enough to allow discharge from ICU (3.78 [2.86;4.25] vs 4.96 [4.41;8.16] μg/ml (p < 0.01) and 2.5 [1.7;3.5] vs 3.8 [2.45;7.8] μg/ml respectively (p < 0.01), Figures 1 and 2). There were no differences in total and HMW adiponectin concentrations between day one and two of the sepsis episode (p = 0.73 and p = 0.46 respectively).

**Figure 3:**
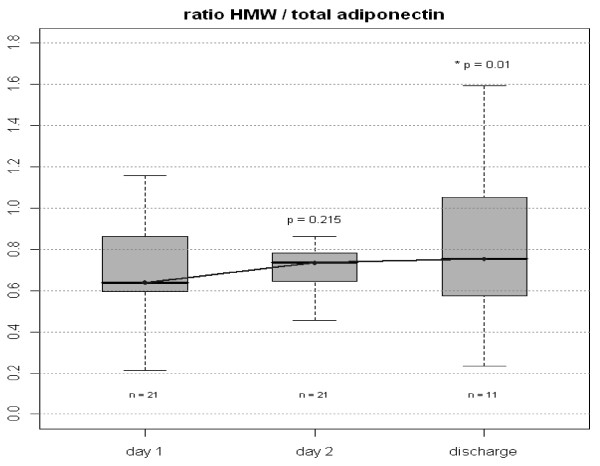
**Ratio of HMW: Total plasma Adiponectin in all patients on day 1, 2 and discharge.** Figure shows median and interquartile range (grey box). Comparisons were made between Day 1 and Day 2 and between Day 2 and Day of Discharge. Wilcoxon test for paired data was used. P values before Bonferroni correction are given in the graph. Corrected p value for Day 2 versus Day of Discharge is 0.02.

The HMW/total adiponectin ratio increased during the observation period (Figure 3). This indicates that formation of HMW adiponectin is suppressed more efficiently in acute sepsis than production of other circulating forms of adiponectin. Total adiponectin plasma concentrations at day 1 correlated with APACHE II values as a marker of severity of disease, while HMW adiponectin levels correlated with CRP concentrations as a marker of the severity of the inflammatory response (Table 3).

**Table 3:** **Correlation between adiponectin, ZAG and severity of disease**

	APACHE II (n = 21)	WCC (n = 21)	CRP (n = 18)
**HMW adiponectin day 1**	0.371 (p = 0.098)	0.049 (p = 0.833)	−0.565 (p = 0.014)
**HMW adiponectin day 2**	0.415 (p = 0.062)	0.173 (p = 0.453)	−0.370 (p = 0.131)
**Total adiponectin day 1**	0.503 (p = 0.020)	0.042 (p = 0.858)	−0.126 (p = 0.618)
**Total adiponectin day 2**	0.415 (p = 0.061)	0.084 (p = 0.716)	−0.229 (p = 0.360)
**HMW/total Adiponectin day 1**	−0.142 (p = 0.539)	−0.064 (p = 0.784)	−0.447 (p = 0.063)
**HMW/total Adiponectin day 2**	−0.267 (p = 0.242)	−0.023 (p = 0.920)	−0.272 (p = 0.276)
**ZAG day 1**	0.237 (p = 0.301)	0.362 (p = 0.106)	0.371 (p = 0.130)
**ZAG day 2**	0.249 (p = 0.277)	0.371 (p = 0.098)	0.463 (p = 0.053)

Correlations between adiponectin and ZAG concentrations respectively and severity of disease and inflammatory response. Spearman rank test was used for all correlations.

HMW adiponectin levels at discharge were higher in women than men (7.7 [3.25; 8.1] vs 2.85 [2.05; 3.625] μg/ml, p = 0.036) which is consistent with the current literature [24–27]. During the acute illness, both sexes had similar HMW and total adiponectin concentrations (males 2.15 [1.43; 3.95], females 2.7 [1.85; 3] μg/ml, p = 0.97). However, on recovery the concentration of HMW in women had increased significantly (p = 0.02) whereas in men it remained unchanged (p = 0.7).

### Serum concentrations of ZAG and correlation with adiponectin levels

**Figure 4:**
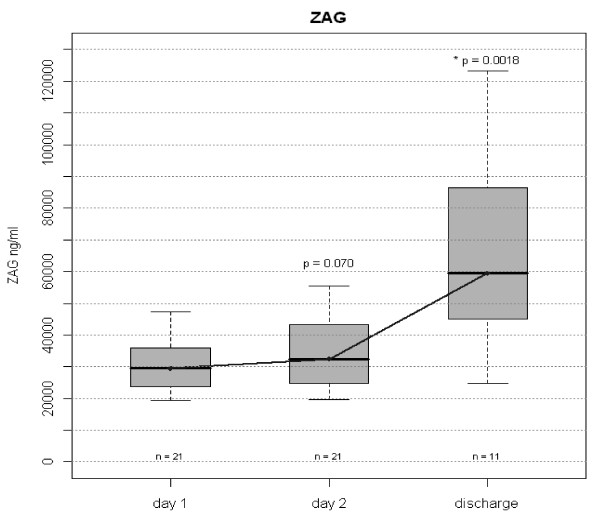
**Plasma ZAG in all patients on day 1, 2 and discharge.** ZAG serum concentrations as measured by ELISA. Figure shows median and interquartile range (grey box). Wilcoxon test for paired data was used. Comparisons were made between Day 1 and Day 2 and between Day 2 and Day of Discharge. P values before Bonferroni correction are given in the graph. Corrected p value for Day 2 versus Day of Discharge is 0.0036.

**Figure 5:**
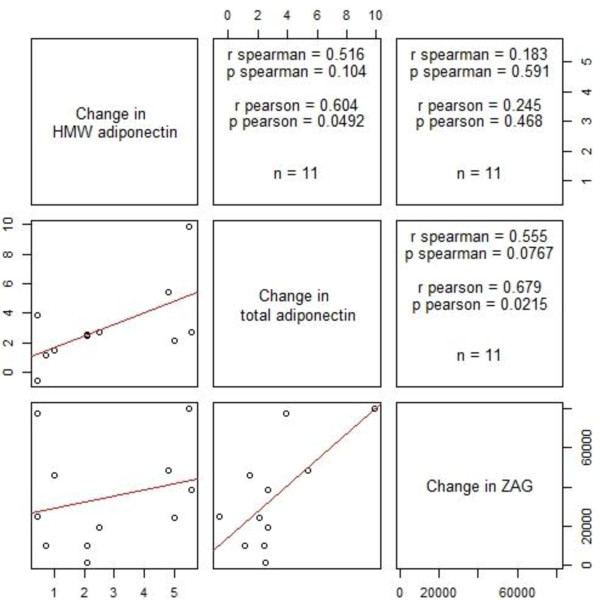
**Correlation between change of HMW adiponectin, total adiponectin and ZAG.** Cutting point between imaginary vertical and horizontal lines show Correlation between change of HMW adiponectin, total adiponectin and ZAG in graphic form on the left and Spearman’s and Pearson’s correlation coefficient on the right cutting point.

Similar to total and HMW adiponectin concentrations, levels of circulating ZAG were significantly lower at day 1 and day 2 of sepsis and increased significantly with an improved clinical condition at discharge from Intensive Care (Figure 4). In patients surviving to discharge (n = 11), a significant correlation between the change in adiponectin (total and HMW) and the change in ZAG serum concentrations was observed (Figure 5). No gender differences were observed for ZAG concentrations on admission or at discharge.

### Comparison between obese and non-obese patients

To evaluate the influence of obesity on severity of disease and degree of systemic inflammation, comparisons were made between patients presenting with a Body Mass Index (BMI) above and below 30 (Table 4). There were no differences ion APACHE II scores, age, and routine laboratory results on admission. No differences were found in serum concentrations of HMW and total adiponectin on admission. Also, ZAG serum concentrations were comparable in both groups. There were also no differences in total and HMW adiponectin levels or ZAG levels between obese and non-obese patients on day 2 and at the time of Intensive Care discharge.

**Table 4:** **Comparison between obese (BMI > 30) and non-obese (BMI < 30) patients**

	BMI < 30					BMI > = 30					
	n	Mean	CI	Median	Range	n	Mean	CI	Median	Range	P value
**Age (Years)**	10	63.2 ± 18.1	[50.3; 76.3]	67	[53.0; 73.3]	11	61.4 9 ± 9.	[54.7; 68.0]	61	[57; 67]	p = 0.622
**APACHE**	10	19.1 ± 5.6	[15.1; 23.1]	20	[15.25; 22.0]	11	22.4 ± 7.1	[17.6; 27.1]	20	[18.5; 28]	p = 0.458
**BMI**	10	23.4 ± 1.5	[22.3; 24.5]	23.85	[22.3; 24.2]	11	34.3 ± 4.8	[31.7; 37.6]	32.1	[30.95; 36.6]	p < 0.001
**WCC (x10/L)**	10	20.5 ± 12.9	[11.2; 29.8]	24.85	[8.8; 30.9]	11	22.8 ± 12.1	[14.7; 31.0]	22.8	[16.25; 29.5]	p = 0.860
**CRP (mg/L)**	8	201.6 ± 95.2.	[122.1; 281.2]	214.5	[174.5; 251.8]	10	196.2 ± 94.9	[128.3; 264.1]	186	[150.25; 199]	p = 0.351
**Hb (g/dL)**	10	10.8 ± 2.54	[9.0; 12.6]	10.5	[8.6; 11.8]	11	9.7 ± 1.7	[8.5; 10.8 ]	10.0	[8.55; 11.2]	p = 0.503
**Creatinine (μmol/L)**	10	149.0 ± 99.7	[77.7; 220.3]	120	[82.0; 198.8]	11	156.6 ± 146.2	[58.3; 254.8]	106	[72.5; 136]	p = 0.833
**Urea (mmol/L)**	10	11.6 ± 6.8	[6.7; 16.5]	10.5	[7.4; 12.9]	11	11.3 ± 6.3	[7.1; 15.5]	8.7	[7.6; 12.7]	p = 0.751
**Bilirubin (μmol/L)**	9	19.3 ± 14.5	[8.2; 30.5]	14	[8.0; 25]	11	36.4 ± 37.7	[11.0; 61.7]	16	[7.5; 70.5]	p = 0.761
**Glucose (mmol/L)**	10	5.8 ± 1.8	[4.5; 7.1]	5.65	[4.4; 6.8]	11	7.4 ± 2.5	[5.7; 9.0]	7	[5.75; 8.4]	p = 0.121
**HMW-Adipo (μg/ml)**	10	2.2 ± 1.3	[1.2; 3.1]	1.75	[1.3; 2.7]	11	2.9 ± 1.0	[2.3; 3.6]	2.9	[2.2; 3.7]	p = 0.130
**Total Adipo (μg/ml)**	10	3.6 ± 1.5	[2.5; 4.7]	3.29	[2.8; 4.2]	11	4.1 ± 1.8	[2.9; 5.3]	3.8	[3.08; 4.3]	p = 0.573
**ZAG (ng/ml)**	10	29473	[22888; 36058]	26490	[23563; 32385]	11	31859.09	[26585; 37133]	30480	[27725; 36223]	p = 0.439

Laboratory and biometric data obtained on admission to Intensive Care comparing patients with body mass index <30 and those with body mass index >30. BMI : Body Mass Index, WCC: white cell count, CRP: C-Reactive protein, Hb: Haemoglobin. HMW Adipo: High Molecular Weight Adiponectin, Total Adipo: Total Adiponectin, ZAG: Zinc-α2-glycoprotein. Parameters are displayed as median, range (IQR), mean ± standard deviation (SD) and 95% confidence intervals (CI) of means.

## Discussion

Adiponectin has been shown to be down-regulated in experimental endotoxaemia. HMW adiponectin, however, has been investigated less, despite some evidence that it is the more active of the different multimers of adiponectin [18, 27]. Our data demonstrate that both HMW adiponectin and total adiponectin concentrations significantly increased between day 1 and day of discharge. This increase in adiponectin was accompanied by a significant clinical improvement in condition, such that intensive care was no longer required. Our results support findings of previous studies demonstrating that total adiponectin increases not only until recovery to discharge, but also up to 8 months after critical illness [28, 29]. Increasing adiponectin concentrations during recovery may reflect resolution of the pro-inflammatory process or a metabolic change indicating a shift towards an anti-inflammatory situation with improved insulin sensitivity.

Emerging literature has demonstrated a greater correlation between markers of insulin resistance and HMW adiponectin rather than the total adiponectin concentration in chronic disease [18, 30], leading to the concept of HMW/total adiponectin ratio as a more sensitive assessment of glucose metabolism. Even without changes in total adiponectin, increases in the ratio reflecting a rise in the HMW adiponectin multimer, confer favourable effects on insulin sensitivity and other metabolic parameters. Therefore, the HMW adiponectin/total adiponectin ratio has been proposed as a useful monitoring tool to assess response drug treatment in Type II DM. In our study the HMW/total adiponectin ratio increased during the observation period, suggesting that the contribution from HMW adiponectin is greater in patients having recovered from sepsis than in patients during their acute stage of sepsis. Patients recruited into this study are a representative sample of patients with sepsis, displaying comparable ages and range of infections to other studies [31–33]. The mean BMI of 29 reflects the national average (28.2) in the UK, hence a representative patient sample was investigated. The correlation between BMI and adiponectin levels has been clearly established in large epidemiological studies. In our study, sample sizes of 10 and 11 patients do not allow conclusions about the impact of body fat on adiponectin levels. The fact that we saw a significant increase in adiponectin concentrations along with clinical recovery, suggests that a massive systemic activation of the immune system as part of the septic response may have a greater impact on serum adiponectin than the obesity status of the patients. Despite the severity of disease (Median APACHE score 20), mean admission glucose levels were below 8 mmol/L with no significant differences between the groups. As part of standard care tight glucose control was achieved by continuous intravenous insulin administration, which together with the methodological difficulties to assess insulin sensitivity in critical illness prevents conclusions about insulin and glucose metabolism in relation to adiponectin changes in our study.

Although the results of this small study require confirmation in a larger patient cohort, a biological role of different adiponectin isoforms in sepsis is likely. The therapeutic potential to modify adiponectin in order to improve recovery and insulin-sensitivity during acute sepsis will have to be explored in future studies. HMW adiponectin concentrations were higher in females than in males at discharge from intensive care which correlates to previous studies [18, 25–27], suggesting a differing regulation of adiponectin between the sexes. This is the first time this has been demonstrated in sepsis. These gender differences were limited to adiponectin concentrations with ZAG levels being similar in both genders on admission as well as at discharge. These findings even in a small patient population indicate that gender differences need to be taken into consideration and that the impact of adiponectin increases on outcome and recovery from critical illness may differ between female and male patients.

In this study we show for the first time that circulating levels of ZAG, an adipokine known to induce cachexia in cancer patients are decreased in acute sepsis and rise during clinical recovery. Furthermore, the course of circulating ZAG concentrations mimics the pattern found for total and HMW adiponectin. We have previously demonstrated that adiponectin production is down-regulated in WAT of mice as part of the inflammatory response to Lipopolysaccharide [21]. These results were confirmed and extended in a recent study demonstrating decreased adiponectin concentrations in mice challenged with LPS [34]. Interestingly, Pini et al. could demonstrate that obese mice showed a greater weight loss seven days after LPS challenge than lean mice.

The results of this single-centre pilot study will have to be confirmed; however, a biological role of different adiponectin isoforms in sepsis appears plausible. In particular, the observed increase of ZAG concentrations during recovery from critical illness should be investigated in a larger patient cohort with an emphasis on a potential correlation with functional data about muscle strength, weight loss and glucose tolerance. The lack of data from patients who did not survive represents a further limitation. Whilst these samples may have given valuable data in patients in whom the inflammatory process did not resolve, for obvious ethical reasons we could not obtain these samples. Due to the small sample size we could not exclude gender, Body Mass Index, age and different aetiologies of sepsis as potential confounders. We did not find significant differences in admission blood results and adipokine levels between survivors and non-survivors. However, the sample size presented here is too small to allow for conclusions about the role of adiponectin in the outcome of sepsis.

## Conclusions

In conclusion, we have demonstrated a significant increase in total and HMW adiponectin and ZAG temporally related to clinical recovery from sepsis. These results encourage further studies investigating the relationship between different adiponectin isoforms and ZAG to explore the therapeutic potential of modulating either of these proteins in sepsis.

### Key messages

- HMW and total adiponectin and ZAG levels are lower on day of admission to ICU compared to day of discharge
- HMW and total adiponectin and ZAG levels significantly increase with clinical recovery from sepsis
- In patients surviving to discharge, there is a significant correlation between change in ZAG and adiponectin concentration

## Abbreviations

APACHE II: Acute physiology and Chronic Health evaluation Score
BSA: Bovine Serum Albumin
CRP: C-reactive protein
HMW: High molecular weight
ICU: Intensive Care Unit
PBS: Phosphate buffered saline
WAT: White adipose tissue
WCC: White cell count
ZAG: Zinc-α2-glycoprotein.

## References

[1] Trayhurn P, Wood IS (2004). Adipokines: inflammation and the pleiotropic role of white adipose tissue. Br J Nutr.

[2] Bing C, Mracek T, Gao D, Trayhurn P (2010). Zinc-alpha2-glycoprotein: an adipokine modulator of body fat mass?. Int J Obes (Lond).

[3] Bing C, Bao Y, Jenkins J, Sanders P, Manieri M, Cinti S, Tisdale MJ, Trayhurn P (2004). Zinc-alpha2-glycoprotein, a lipid mobilizing factor, is expressed in adipocytes and is up-regulated in mice with cancer cachexia. Proc Natl Acad Sci U S A.

[4] Bao Y, Bing C, Hunter L, Jenkins JR, Wabitsch M, Trayhurn P (2005). Zinc-alpha2-glycoprotein, a lipid mobilizing factor, is expressed and secreted by human (SGBS) adipocytes. FEBS Lett.

[5] Gao D, Trayhurn P, Bing C (2010). Macrophage-secreted factors inhibit ZAG expression and secretion by human adipocytes. Mol Cell Endocrinol.

[6] Mracek T, Ding Q, Tzanavari T, Kos K, Pinkney J, Wilding J, Trayhurn P, Bing C (2010). The adipokine zinc-alpha2-glycoprotein (ZAG) is downregulated with fat mass expansion in obesity. Clin Endocrinol (Oxf).

[7] Trayhurn P (2005). Endocrine and signalling role of adipose tissue: new perspectives on fat. Acta Physiol Scand.

[8] Kadowaki T, Yamauchi T (2005). Adiponectin and adiponectin receptors. Endocr Rev.

[9] Berg AH, Combs TP, Du X, Brownlee M, Scherer PE (2001). The adipocyte-secreted protein Acrp30 enhances hepatic insulin action. Nat Med.

[10] Fruebis J, Tsao TS, Javorschi S, Ebbets-Reed D, Erickson MR, Yen FT, Bihain BE, Lodish HF (2001). Proteolytic cleavage product of 30-kDa adipocyte complement-related protein increases fatty acid oxidation in muscle and causes weight loss in mice. Proc Natl Acad Sci U S A.

[11] Maeda N, Shimomura I, Kishida K, Nishizawa H, Matsuda M, Nagaretani H, Furuyama N, Kondo H, Takahashi M, Arita Y, Komuro R, Ouchi N, Kihara S, Tochino Y, Okutomi K, Horie M, Takeda S, Aoyama T, Funahashi T, Matsuzawa Y (2002). Diet-induced insulin resistance in mice lacking adiponectin/ACRP30. Nat Med.

[12] Park PH, Huang H, McMullen MR, Mandal P, Sun L, Nagy LE (2008). Suppression of lipopolysaccharide-stimulated tumor necrosis factor-alpha production by adiponectin is mediated by transcriptional and post-transcriptional mechanisms. J Biol Chem.

[13] Tsuchihashi H, Yamamoto H, Maeda K, Ugi S, Mori T, Shimizu T, Endo Y, Hanasawa K, Tani T (2006). Circulating concentrations of adiponectin, an endogenous lipopolysaccharide neutralizing protein, decrease in rats with polymicrobial sepsis. J Surg Res.

[14] Yamauchi T, Kamon J, Minokoshi Y, Ito Y, Waki H, Uchida S, Yamashita S, Noda M, Kita S, Ueki K, Eto K, Akanuma Y, Froguel P, Foufelle F, Ferre P, Carling D, Kimura S, Nagai R, Kahn BB, Kadowaki T (2002). Adiponectin stimulates glucose utilization and fatty-acid oxidation by activating AMP-activated protein kinase. Nat Med.

[15] Yamauchi T, Kamon J, Waki H, Terauchi Y, Kubota N, Hara K, Mori Y, Ide T, Murakami K, Tsuboyama-Kasaoka N, Ezaki O, Akanuma Y, Gavrilova O, Vinson C, Reitman ML, Kagechika H, Shudo K, Yoda M, Nakano Y, Tobe K, Nagai R, Kimura S, Tomita M, Froguel P, Kadowaki T (2001). The fat-derived hormone adiponectin reverses insulin resistance associated with both lipoatrophy and obesity. Nat Med.

[16] Yokota T, Oritani K, Takahashi I, Ishikawa J, Matsuyama A, Ouchi N, Kihara S, Funahashi T, Tenner AJ, Tomiyama Y, Matsuzawa Y (2000). Adiponectin, a new member of the family of soluble defense collagens, negatively regulates the growth of myelomonocytic progenitors and the functions of macrophages. Blood.

[17] Wang Y, Lam KS, Yau MH, Xu A (2008). Post-translational modifications of adiponectin: mechanisms and functional implications. Biochem J.

[18] Hara K, Horikoshi M, Yamauchi T, Yago H, Miyazaki O, Ebinuma H, Imai Y, Nagai R, Kadowaki T (2006). Measurement of the high-molecular weight form of adiponectin in plasma is useful for the prediction of insulin resistance and metabolic syndrome. Diabetes Care.

[19] Nakano Y, Tobe T, Choi-Miura NH, Mazda T, Tomita M (1996). Isolation and characterization of GBP28, a novel gelatin-binding protein purified from human plasma. J Biochem.

[20] Pajvani UB, Du X, Combs TP, Berg AH, Rajala MW, Schulthess T, Engel J, Brownlee M, Scherer PE (2003). Structure-function studies of the adipocyte-secreted hormone Acrp30/adiponectin: Implications fpr metabolic regulation and bioactivity. J Biol Chem.

[21] Leuwer M, Welters I, Marx G, Rushton A, Bao H, Hunter L, Trayhurn P (2009). Endotoxaemia leads to major increases in inflammatory adipokine gene expression in white adipose tissue of mice. Pflugers Arch.

[22] Vassiliadi DA, Tzanela M, Kotanidou A, Orfanos SE, Nikitas N, Armaganidis A, Koutsilieris M, Roussos C, Tsagarakis S, Dimopoulou I (2012). Serial changes in adiponectin and resistin in critically ill patients with sepsis: associations with sepsis phase, severity, and circulating cytokine levels. J Crit Care.

[23] Levy MM, Fink MP, Marshall JC, Abraham E, Angus D, Cook D, Cohen J, Opal SM, Vincent JL, Ramsay G (2003). 2001 SCCM/ESICM/ACCP/ATS/SIS International Sepsis Definitions Conference. Crit Care Med.

[24] Almeda-Valdes P, Cuevas-Ramos D, Mehta R, Gomez-Perez FJ, Cruz-Bautista I, Arellano-Campos O, Navarrete-Lopez M, Aguilar-Salinas CA (2010). Total and high molecular weight adiponectin have similar utility for the identification of insulin resistance. Cardiovasc Diabetol.

[25] Fujimatsu D, Kotooka N, Inoue T, Nishiyama M, Node K (2009). Association between high molecular weight adiponectin levels and metabolic parameters. J Atheroscler Thromb.

[26] Lara-Castro C, Luo N, Wallace P, Klein RL, Garvey WT (2006). Adiponectin multimeric complexes and the metabolic syndrome trait cluster. Diabetes.

[27] Pajvani UB, Hawkins M, Combs TP, Rajala MW, Doebber T, Berger JP, Wagner JA, Wu M, Knopps A, Xiang AH, Utzschneider KM, Kahn SE, Olefsky JM, Buchanan TA, Scherer PE (2004). Complex distribution, not absolute amount of adiponectin, correlates with thiazolidinedione-mediated improvement in insulin sensitivity. J Biol Chem.

[28] Langouche L, Vander Perre S, Wouters PJ, D'Hoore A, Hansen TK, Van den Berghe G (2007). Effect of intensive insulin therapy on insulin sensitivity in the critically ill. J Clin Endocrinol Metab.

[29] Jernas M, Olsson B, Sjoholm K, Sjogren A, Rudemo M, Nellgard B, Carlsson LM, Sjostrom CD (2009). Changes in adipose tissue gene expression and plasma levels of adipokines and acute-phase proteins in patients with critical illness. Metabolism.

[30] Lara-Castro C, Doud EC, Tapia PC, Munoz AJ, Fernandez JR, Hunter GR, Gower BA, Garvey WT (2008). Adiponectin multimers and metabolic syndrome traits: relative adiponectin resistance in African Americans. Obesity.

[31] Alberti C, Brun-Buisson C, Burchardi H, Martin C, Goodman S, Artigas A, Sicignano A, Palazzo M, Moreno R, Boulme R, Lepage E, Le Gall R (2002). Epidemiology of sepsis and infection in ICU patients from an international multicentre cohort study. Intensive Care Med.

[32] Angus DC, Linde-Zwirble WT, Lidicker J, Clermont G, Carcillo J, Pinsky MR (2001). Epidemiology of severe sepsis in the United States: analysis of incidence, outcome, and associated costs of care. Crit Care Med.

[33] Martin GS, Mannino DM, Eaton S, Moss M (2003). The epidemiology of sepsis in the United States from 1979 through 2000. N Engl J Med.

[34] Pini M, Castellanos KJ, Rhodes DH, Fantuzzi G (2013). Obesity and IL-6 interact in modulating the response to endotoxemia in mice. Cytokine.

[CR35] The pre-publication history for this paper can be accessed here: http://www.biomedcentral.com/1471-2253/14/124/prepub

